# Damaged Insula Network Contributes to Depression in Parkinson’s Disease

**DOI:** 10.3389/fpsyt.2020.00119

**Published:** 2020-03-10

**Authors:** Peiyu Huang, Xiaojun Guan, Tao Guo, Qiaoling Zeng, Min Xuan, Quanquan Gu, Xiaojun Xu, Cheng Zhou, Jingjing Wu, Minming Zhang

**Affiliations:** Department of Radiology, 2^nd^ Affiliated Hospital, Zhejiang University School of Medicine, Hangzhou, China

**Keywords:** Parkinson’s disease, depression, insula, functional connectivity, magnetic resonance imaging

## Abstract

**Background:**

Depression is common in patients with Parkinson’s disease (PD). Our previous studies suggest that depressed PD patients have altered insula structures. It is, however, still unknown whether the altered structures cause disruption of insula functional networks, further contributing to depression in PD.

**Methods:**

In the present study, 17 depressed PD patients, 17 non-depressed PD patients, and 17 normal controls were enrolled. All subjects went through neurological and psychiatric clinical assessments. Resting-state functional magnetic resonance imaging and seed-based insula functional analyses were performed to examine the insula functional connectivity alterations in PD patients.

**Results:**

We found that compared with normal controls, PD patients exhibited significantly decreased insula functional connectivity widely across the whole brain. Compared with non-depressed PD patients, depressed patients showed further decreased functional connectivity in the middle frontal gyrus and inferior parietal lobe. Furthermore, connectivity between the left anterior insula and middle frontal gyrus was positively correlated with the cognitive scale score.

**Conclusion:**

These results suggest that insula networks were severely damaged in PD patients, and that the disrupted connection between the salience network and executive control network might contribute to depression in PD.

## Introduction

Parkinson’s disease (PD) is a progressive neurodegenerative disorder of the central nervous system, characterized by motor symptoms such as tremor, bradykinesia and rigidity. Depression is one of the most common non-motor symptoms (1) in patients with Parkinson’s disease (PD). The prevalence of depression in PD is high (2–4). Depression may precede the clinical onset of PD, and last throughout the duration of the disease. It can severely lower the living quality of patients and be an economic burden to the families of patients and society (5). Understanding the neural mechanism of depression in PD is important for clinical diagnosis and treatment.

During PD development, the dopamine, serotonin, and noradrenaline neurotransmitter systems are damaged due to Lewy body accumulation in the mid-brain nucleus (6). As these neurotransmitters are vital for the regulation of emotion and cognitive functions, degeneration may cause emotional disturbances (7). Furthermore, the Lewy body accumulation gradually spreads from the subcortical structures to cortical regions, causing loss of neurons in brain areas such as the amygdala and hippocampus, which are closely related to emotional functions (8, 9). These pathological changes have been related to depression in previous studies (8, 9). Depression is therefore considered to be intrinsic to PD rather than a reactive feature (10).

Our previous studies demonstrated that depressed PD patients had decreased gray matter area and disrupted white matter integrity in the insula (11, 12). The insula is a part of the cerebral cortex located deep within the lateral sulcus, which is believed to be involved in a variety of brain functions (13) including perception, emotion, cognition, etc. Damaged insula structure and function have been consistently reported to be related to various PD non-motor symptoms (14, 15), including cognitive impairments, affective disorders, autonomic dysfunction, etc. Christopher et al. summarized the literature and proposed that the insula processes various sensory input information (16), and is responsible for generating subjective feelings. Therefore, a disruption of the insula’s ability to receive or integrate information may alter the related brain functions and then behaviors in PD patients. As depression is a “disconnection” syndrome (17), it is necessary to explore the integrity of insula related brain networks in PD patients and its relationship to depression. In particular, because different insula sub-regions are involved in different brain functions, they may contribute differently to depression in PD.

In the present study, we aim to investigate whether the disruption of insula related brain networks contributes to depression in PD. Resting-state functional magnetic resonance imaging and seed-based functional connectivity (FC) analyses were introduced to investigate this question. We expected to find decreased insula FC within brain areas related to cognitive and emotional regulations in depressed PD patients.

## Materials and Methods

### Subjects

Two groups of PD patients (with and without depression, 17 for each group) were recruited from the Department of Neurology, Second Affiliated Hospital of Zhejiang University. The PD diagnosis was made by a senior neurologist according to the UK PD Brain Bank criteria. Unipolar depression was diagnosed by an experienced psychiatrist according to the Diagnostic and Statistical Manual of Mental Disorders, 4th Edition criteria (DSM-IV). Scores of the Unified Parkinson’s Disease Rating Scale (UPDRS), the Hamilton Rating Scale for Depression (HRSD), Hamilton Anxiety Rating Scale (HAMA) and the Mini-Mental State Examination (MMSE) were obtained from all subjects. **Table 1** shows detailed characteristics of the two groups. Patients were excluded from the study if they had: (1) cerebrovascular disorders, including a previous stroke, a history of seizure, hydrocephalus, an intracranial mass, a history of head injury, previous neurological surgery, or other neurological diseases; (2) any current DSM-IV Axis I diagnosis other than MDD, as determined by an experienced psychiatrist; (3) treatment with antidepressants or other psychiatric therapy; or (4) dementia. For technical reasons, participants who were unable to keep still during the MRI because of head motion were not eligible to be in the study. Seventeen normal controls (NC) matched for age and sex were also enrolled. All subjects provided written informed consent prior to the study. The research was approved by the medical ethics committee of the Second Afﬁliated Hospital, Zhejiang University School of Medicine. All clinical investigations were conducted in accordance to the principles expressed in the Declaration of Helsinki.

**Table 1:** Demographic characteristics.

Index	dPD	ndPD	NC	Test value	*p* value
Gender (m/f)	8/9	9/8	9/8	0.157^###^	0.925
Age, y, mean ± SD	59.4± 8.9	59.1 ± 9.9	59.18 ± 9.95	0.004^##^	0.996
Duration, mean ± SD	3.6 ± 3.3	4.3 ± 3.7	–	-0.565^#^	0.576
Hoehn and Yahr stage, median	2.5	2.5	–	3.159^###^	0.206
UPDRS, mean ± SD	44.1 ± 12.3	40.5 ± 9.2	–	0.962^#^	0.343
MMSE, mean ± SD	26.2 ± 2.5	26.1 ± 2.7	–	0.131^#^	0.897
HRSD, mean ± SD	30.0 ± 6.1	8.2± 5.6	–	6.125^#^	0.000
HAMA, mean ± SD	16.1 ± 8.1	7.1 ± 6.8		3.501^#^	0.001

^#^t value; ^##^F value; ^###^Pearson chi-square.

dPD, depressed PD; ndPD, non-depressed PD; NC, normal controls.

### Image Acquisition

All the scans were performed on a 3.0T GE MR scanner in the Department of Radiology, Second Affiliated Hospital, Zhejiang University School of Medicine. Ear plugs and foam pads were used to reduce noise and head motion. Blood oxygenation level dependent (BOLD) images were acquired using an echo planar imaging sequence (TR = 2000ms, TE = 30ms, flip angle = 90°, FOV = 240 × 240mm^2^, matrix = 64 × 64, slice thickness = 5 mm; slice gap = 1 mm; 23 interleaved axial slices). A total of 185 resting-state BOLD images were acquired from each subject. Anatomical images were acquired after the functional imaging, and consisted of a 3-D GRE T1-weighted sequence (TR/TE = 5.14ms/1.17 ms, FOV = 240 × 240mm^2^, matrix = 256 × 256, slice thickness = 1.2mm; no gap, 124 sagittal slices). Several other sequences were also acquired, including a T2-weighted imaging sequence, a diffusion tensor imaging sequence, and a susceptibility weighted imaging sequence. A total scan took 40 min per subject. Before scanning, subjects were instructed to relax with their eyes closed without falling asleep, and without directed systematic thought. This was confirmed after completion of scanning.

### Image Processing

Preprocessing was performed using Data Processing Assistant for Resting-State fMRI [DPARSF(18), by YAN Chao-Gan, http://www.restfmri.net] and Resting-State fMRI Data Analysis Toolkit [REST V1.8, (19), http://www.restfmri.net]. The first 10 images were excluded from the analysis. The remaining images were corrected for slice timing using the middle slice as a reference, realigned to remove head motion. Several nuisance covariates, including the Friston-24 head motion parameters [six head motion parameters, six head motion parameters from the previous time point, and the 12 corresponding squared items (20)], as well as white matter and CSF signals (calculated using the CompCor method (21), within the mask from the segmentation of co-registered T1 images), were regressed out to improve signal-noise ratio and minimize the motion artifact. The resulted images were normalized into the standard space using DARTEL and resampled to 3 × 3 × 3 mm voxel size. Then we smoothed the images using a 6-mm Gaussian kernel, removed the linear trend, and performed bandpass filtering (0.01~0.1Hz).

During preprocessing, to further minimize the influence of head motion on the functional connectivity calculation, subjects having head motion with >2 mm of translation or 2° of rotation were excluded. Framewise displacement (FD) (22) of head movement was evaluated with the sum of the absolute values of the six motion parameter derivatives. Using FD, we censored each subjects’ resting-state BOLD time series that were associated with sudden head motion and excluded the time point where FD > 0.5mm. After this scrubbing procedure, subjects whose total length of data were <130 were also excluded. No subject was excluded due to head motion in the present study.

### Functional Connectivity Analysis

According to structural or functional organization, the insula can be divided into two, three or more sub-regions. Previously, many studies have demonstrated the distinct functions of different sub-regions (23, 24). In general, the anterior insula is more related to cognitive and emotional functions, while the posterior insula contributes more to autonomic and sensory functions. In our previous studies, we found that fiber connections were disrupted near the left anterior insula. Therefore, here we have chosen four regions of interest (ROI): left/right and anterior/posterior insula. The ROI was constructed from the Human Brainnetome Atlas (HBA) (25), which was created based on the tractography results of Human Connectome Project data. Insula was divided into six sub-regions in the HBA. Here the anterior insula mask was constructed by combining the INS_L(R)_6_2, INS_L(R)_6_3 and INS_L(R)_6_6 areas, and the posterior insula mask was constructed by combining the INS_L(R)_6_1, INS_L(R)_6_4, INS_L(R)_6_5 areas. The resulting masks showed good overlapping with the anterior/posterior divisions in previous literature. These ROIs were then used as seed regions for seed-based FC analysis. Pearson’s correlation was calculated between the average time series within the ROIs and other brain areas. Lastly, the resulting connectivity maps were transformed to Z maps using Fisher r-to-z transformation.

### Statistical Analysis

#### Demographic Data

Age was compared among the three groups using one-way analysis of variance (ANOVA). A chi-square test was performed to compare the sex differences. Disease duration and scale scores were compared between the two PD groups using two-sample t-test.

#### Image Data

FD was compared among the three groups using one-way ANOVA. First, a one-sample t-test was used to demonstrate the connectivity patterns in each group. The results showed similar insula FC patterns to previous studies. Then, general linear models were used to compare functional connectivity maps among the three groups, with age and sex used as covariates. To identify whether PD patients had lower insula functional connectivity, all PD subjects were first pooled together and compared with the NC group. Comparison between the depressed and non-depressed PD patients were then made to demonstrate depression-related changes. The threshold for control-PD comparison was set at *p* < 0.05, and corrected using the FDR (false discovery rate) method. The threshold for comparison between the two PD groups was set at *p* < 0.001, and corrected using Gaussian Random Field theory. Signals from the significant clusters were also extracted to further explore the relationship between insula connectivity and scale scores (partial correlation, age and sex controlled).

## Results

Statistical analysis showed no age and sex differences among the three groups (**Table 1**). Disease characteristics such as duration, Hoehn and Yahr Scale, UPDRS and MMSE scores also showed no difference between the two PD groups. The depressed PD patients had higher HRSD and HAMA scores compared to the non-depressed PD group.

There was no significant difference of FD among the three groups. After removing the timepoints with FD > 0.5, the remaining number of images in the BOLD data also had no difference among the three groups.

**Figure 1:**
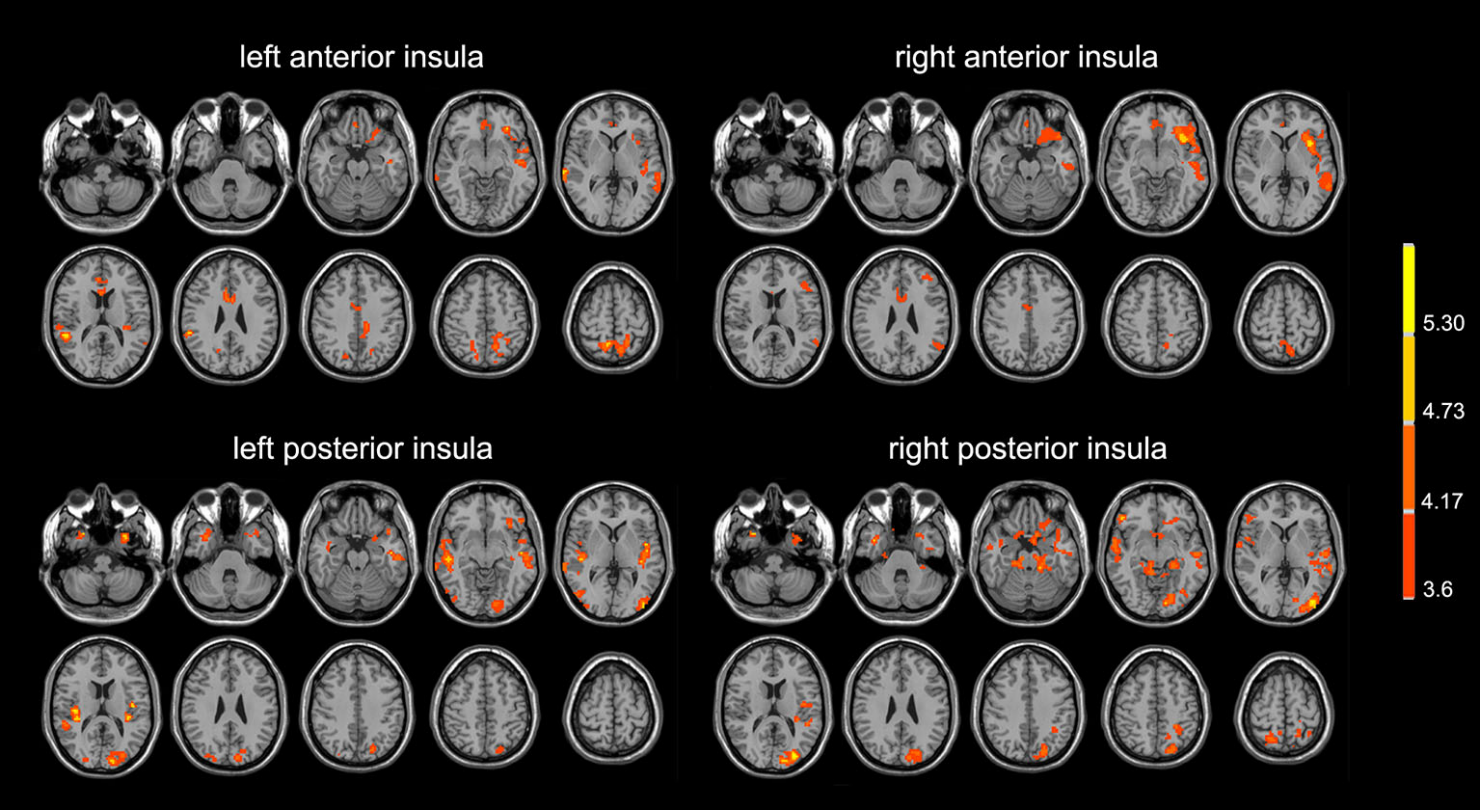
PD patients showed lower insula functional connectivity compared with normal controls. The images were displayed in radiological conventions.

**Figure 2:**
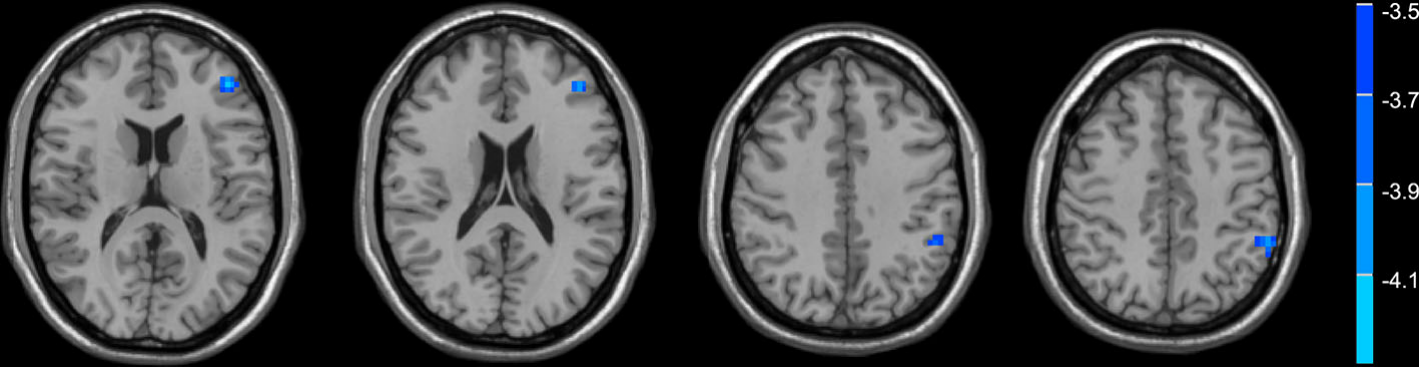
Depressed PD patients had lower functional connectivity between the left anterior insula and frontal-parietal regions compared with non-depressed patients.

Compared with normal controls, the PD patients showed widely decreased insula FC in the whole brain (**Table 2**, **Figure 1**). In general, the anterior insula’s connection with the fronto-parietal network, cingulate gyrus, and temporal lobe significantly decreased, while the posterior insula had more decreased FC within the temporal lobe, sensorimotor cortex, parietal, and occipital lobe. The decreased FC was not limited to the ipsilateral hemisphere, but also extended to the contralateral hemisphere. Compared with non-depressed patients, the depressed PD patients showed decreased FC (**Table 3**, **Figure 2**) between left anterior insula and left middle frontal gyrus (MFG), left inferior parietal lobe. FC between left anterior insula and left MFG showed significant correlation with MMSE scores (*r *= 0.522, *p *= 0.046). No other correlations were found between the FCs and scales scores.

**Table 2:** PD patients showed lower insula functional connectivity compared with normal controls.

Cluster Size	Hemisphere	Brain Region	MNI coordinate	Peak t
Left anterior insula				
150	L/R	Anterior cingulate	3 39 -18	5.3376
437	L	Superior temporal gyrus Inferior frontal gyrus	-48 -15 -15	5.6031
257	R	Superior temporal gyrus Inferior parietal lobe	51 -42 15	5.4573
402	L	Superior temporal gyrus	-60 -51 6	4.5668
217	R	Middle cingulate	9 3 33	5.3686
631	L/R	Precuneus	-12 -63 60	4.9049
Left Posterior Insula				
744	L	Superior/middle temporal gyrus Inferior frontal gyrus	-45 -15 9	5.3863
750	R	Superior/middle temporal gyrus Insula Precentral gyrus	33 9 -36	5.47
118	L	Lingual gyrus	-12 -87 -9	4.0507
132	R	Middle occipital gyrus	51 -75 3	4.026
110	L	Middle occipital gyrus	-42 -90 0	4.6682
298	L	Superior occipital gyrus	-15 -93 18	4.7786
112	R	Superior occipital gyrus Cuneus	30 -87 33	4.2613
Right Anterior Insula				
1478	L	Middle/Inferior frontal gyrus Superior/middle/inferior temporal gyrus Parietal lobe Insula	-39 15 -3	6.987
117	R	Medial frontal gyrus	3 39 -15	6.1421
132	L/R	Anterior/middle cingulate gyrus	6 -3 36	5.3245
160	L/R	Precuneus Superior parietal lobe	-6 -63 60	4.7324
Right Posterior Insula				
380	L	Middle/inferior frontal gyrus Superior/middle temporal gyrus	-21 12 -33	4.7062
124	R	Superior/middle temporal gyrus	30 12 -42	5.1911
245	L	Hippocampus/parahippocampal gyrus	-24 -33 -15	5.1972
225	R	Parahippocampal gyrus Lingual gyrus	12 -24 -18	4.8424
222	R	Superior/middle temporal gyrus	60 -6 -15	5.0978
125	R	Inferior frontal gyrus	51 36 -9	4.9634
990	L	Middle occipital gyrus Superior/inferior parietal lobe Cuneus	-39 -87 0	5.886
526	L	Superior/middle temporal gyrus	-33 -33 12	5.0295
195	L	Superior/inferior parietal lobe Precuneus	-18 -54 57	5.079
122	R	Superior/inferior parietal lobe precuneus	18 -60 60	4.9414

MNI, Montreal Neurological Institute; L, left; R, right.

**Table 3:** Depressed PD patients had lower functional connectivity between the left anterior insula and frontal-parietal regions compared with non-depressed patients.

Cluster Size	Hemisphere	Brain Region	MNI coordinate	Peak t
62	L	middle frontal gyrus	-42 42 18	-3.6645
30	L	inferior parietal lobe	-54 -42 42	-3.9734

MNI, Montreal Neurological Institute; L, left; R, right.

## Discussion

In the present study, we compared insula FC among the normal controls, depressed, and non-depressed PD patients. We aimed to investigate whether the disruption of insula FC contributed to depression in PD. We found that PD patients had significantly lower insula FC compared with normal controls, and that depressed patients showed much lower insula FC than the non-depressed patients in the fronto-parietal regions. Furthermore, the connectivity strength between insula and MFG showed a significant correlation with MMSE scores.

First, PD patients exhibited widely decreased insula FC with many brain areas. It is possible that insula functions had been damaged in these patients. According to Braak’s pathological study, the insula may be fully involved with the Lewy body during Braak 5-6 stage (26). However, recent evidence supports that insula damage may happen even earlier (27). In several morphometry studies, gray matter volume reduction is consistently found in the insula of PD patients (28, 29). These findings were confirmed by a meta-analysis on 17 studies (14). Decreased regional cerebral blood flow (30), reduced serotonin (31), and dopamine transporter (32) binding was also found in PD patients. On the other hand, this deceased FC could also be the results of widely disrupted fiber connections in PD patients. Several main association fibers, such as superior longitudinal fasciculus and inferior longitudinal fasciculus, have been found damaged in PD patients (33). Notably, insula FC changes were not limited to the ipsilateral hemisphere, but also extended to contralateral hemispheres, indicating reduced inter-hemispheric connections.

We found that depressed PD patients had decreased left anterior insula FC with the left MFG, and inferior parietal lobe compared with non-depressed patients. As mentioned above, different sub-regions of insula may participate in different brain functions. The anterior insula has fiber connections with the ventral striatum, the anterior cingulate gyrus, the temporal lobe and frontal lobe (34), and is related to various emotional and cognitive functions in the human brain. The disrupted anterior insula FC may therefore cause disturbances in these brain functions. Previously, we had demonstrated decreased gray matter area and disrupted white matter integrity in the same region (11, 12), but no correlation was found between imaging findings and behavioral scores in both studies. Therefore, we had speculated that those structural deficits could be acting indirectly.

We didn’t find any significant correlation between insula FC and depression scores. The FC between the left anterior insula and MFG was, however, correlated with MMSE scores, indicating its contribution to cognitive impairment in PD patients. This finding is consistent with several recent studies (35, 36) using the independent component analysis method to study the relationship between cognition and large brain network connectivity in PD patients. It is possible that insula network degeneration impaired cognitive functions and increased vulnerability to depression in these patients. It corresponds well with the previous clinical observations that depression is strongly related to cognitive decline in PD patients (37). However, while our two PD groups had similar MMSE scores, one group did not have depression. We speculate that other influencing factors, such as genetic variations (38) and social status (39) might have interacted with cognitive impairment and caused depression. In addition to insula network dysfunction, brain structural and functional alterations in other brain regions and networks may also have a superimposed effect. But this is beyond the scope of the current study and may be explored in future research.

From a large-scale network perspective, the anterior insula is the key node of an important network called the salience network (SN). The SN is well known for its function in processing salience information (40) and regulating balance between different large scale networks (41). Furthermore, the frontal-parietal areas found here belongs to the left executive control network (ECN) (42), which is involved in multiple cognitive processes, such as attention, language, and memory. Therefore, these findings may suggest that the interaction between the SN and ECN had been damaged in depressed PD patients. This obstructed communication may cause disrupted top-down cognitive modulation over the subcortical structure, giving rise to depression. Previously, this mechanism has also been reported (43) in patients with major depressive disorder (MDD). Therefore, our results may suggest that impaired interactions between large-scale brain networks are critical for the occurrence of PD depression.

There are several limitations in the present study. The first is the anxiety levels of the PD patients. Because of the common existence of anxiety in PD patients and limited patient resources, the anxiety levels of PD groups were difficult to match, which may be confounding to the study results. The second limitation is the small sample size. Nevertheless, our results are consistent with previous studies and suggest that the insula is an important brain region in PD depression.

In general, these results confirmed our hypothesis that PD patients have disrupted insula FC and it contributes to the occurrence of depression. Indeed, PD depression has very complex pathophysiological origins, involving multiple neuro-transmitter systems and widespread brain regions (44). Our results reflect this complexity, highlighting the role of disrupted insula connectivity. As insula has multiple neuro-transmitter receptors, different drugs may restore different insula circuits (as well as other brain networks) and relieve depressive symptoms. Future studies may be performed to further confirm the insula’s role in PD depression, and to investigate whether pharmacological interventions or non-invasive brain stimulations could recover insula connectivity and improve depressive symptoms.

## Data Availability Statement

The datasets generated for this study are available on request to the corresponding author.

## Ethics Statement

The studies involving human participants were reviewed and approved by the medical ethics committee of the Second Afﬁliated Hospital, Zhejiang University School of Medicine. The patients/participants provided their written informed consent to participate in this study.

## Author Contributions

XG, QG, MX, TG, CZ, JW, XX, and QZ enrolled the patients and performed data acquisition. PH analyzed the data and wrote the paper. MZ directed the research. All authors reviewed the manuscript.

## Funding

This study was supported by the 13th Five-year Plan for National Key Research and Development Program of China (Grant No. 2016YFC1306600), the National Natural Science Foundation of China (Grant Nos. 81771820 and 81971577), and the Natural Science Foundation of Zhejiang Province (Grant No. LSZ19H180001). They had no further role in the study design, data collection, data analysis, and paper writing.

## Conflict of Interest

The authors declare that the research was conducted in the absence of any commercial or financial relationships that could be construed as a potential conflict of interest.
